# New Technique of Exposed Glaucoma Drainage Tube Repair: Report of a Case

**DOI:** 10.5005/jp-journals-10008-1185

**Published:** 2015-09-25

**Authors:** Tamara L Berezina, Robert D Fechtner, Amir Cohen, Eliott E Kim, David S Chu

**Affiliations:** Research Associate, Department of Ophthalmology, Institute of Ophthalmology and Visual Science, Rutgers New Jersey Medical School New Jersey, United States; Professor, Department of Ophthalmology, Institute of Ophthalmology and Visual Science, Rutgers New Jersey Medical School New Jersey, United States; Assistant Professor, Department of Ophthalmology, Institute of Ophthalmology and Visual Science, Rutgers New Jersey Medical School New Jersey, United States; Summer Research Intern, Department of Ophthalmology, Institute of Ophthalmology and Visual Science, Rutgers New Jersey Medical School New Jersey, United States; Clinical Associate Professor, Department of Ophthalmology, Institute of Ophthalmology and Visual Science, Rutgers New Jersey Medical School New Jersey, United States

**Keywords:** Conjunctiva deficiency, Corneal patch graft, Glaucoma drainage device, Keratoglobus, Wound healing.

## Abstract

We present the case of successful repair of an exposed glaucoma drainage tube by cornea graft fixation with tissue adhesive, and without subsequent coverage by adjacent conjunctiva or donor tissues. Patient with history of keratoglobus with thin cornea and sclera, and phthisical contralateral eye, underwent three unsuccessful corneal grafts followed by Boston type 1 keratoprosthesis in the right eye. Ahmed drainage device with sclera patch graft was implanted to control the intraocular pressure. Two years later the tube eroded through sclera graft and conjunctiva. Repair was performed by covering the tube with a corneal patch graft secured by tissue adhesive after the conjunctiva in this area was dissected away. The cornea graft was left uncovered due to fragility of adjacent conjunctiva. The healing of ocular and graft surfaces was complete prior to the 1 month follow-up. Conjunctival epithelium covered the corneal patch graft. At 12 months follow-up, the graft and the tube remained stable. Our report suggests that corneal patch graft fixation to the sclera by means of tissue adhesive, without closing the conjunctiva, can be considered as an effective alternative surgical approach for managing exposed glaucoma drainage tube, accompanied by adjacent conjunctiva tissue deficiency.

**How to cite this article:** Berezina TL, Fechtner RD, Cohen A, Kim EE, Chu DS. New Technique of Exposed Glaucoma Drainage Tube Repair: Report of a Case. J Curr Glaucoma Pract 2015;9(2):62-64.

## INTRODUCTION

Erosion of the tube through graft material and conjunctiva, after glaucoma drainage device surgery, is a serious complication that may lead to local infection and endophthalmitis. The frequency of erosion varies from 2.5 to 7%.^1-5^ In patients with Boston keratoprosthesis and glaucoma drainage device, it is reported from 14.6 to more than 50% of cases.^6,7^ Revision surgery commonly involves covering the exposed tube with patch grafts of materials, such as scleral, cornea, pericardium, or other donor tissues, and advancing conjunctiva over the graft. This technique may be challenging in eyes that previously underwent multiple surgeries because of scar formation and partial immobility of conjunctival tissue. The aim of this publication is to present a novel way of repairing an exposed glaucoma drainage tube when deficiency and fragility of conjunctiva prevented coverage over the patch.

## MATERIALS AND METHODS

### History of the Disease

**Figs 1A to F:**
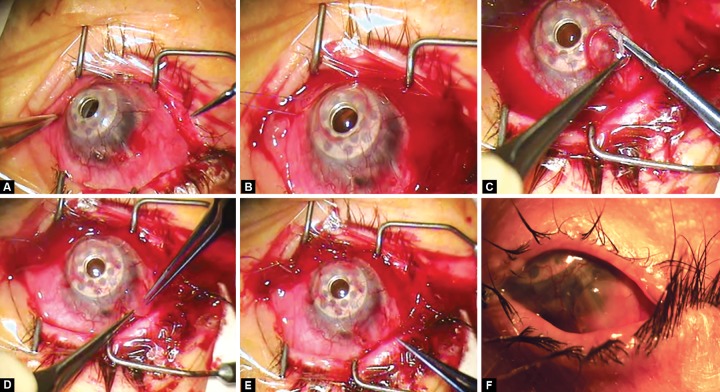
Clinical photographs showing the surgical technique of exposed glaucoma drainage tube repair: (A) exposed tube prior to the procedure, (B) conjunctiva around and under the tube excised, (C) trimming of edge of cornea patch graft, (D) securing graft with tissue glue, (E) tube is covered postoperatively, conjunctiva retracted, (F) one month after procedure showing epithelialized graft (surgeon’s view)

A 57-year-old patient with keratoglobus and phthisical left eye underwent three unsuccessful corneal grafts, followed by Boston type 1 keratoprosthesis and Ahmed drainage device with sclera patch graft in the right eye. Glaucoma drainage implant plate was placed 9 mm posterior to the corneal limbus and fixed to the sclera using 8-0 nylon sutures. The tube was passed through a short scleral tunnel just posterior to the corneal limbus. The anterior extraocular portion of the tube was covered with preserved human donor scleral graft that was secured to host sclera with 10-0 nylon suture. At 24 months after glaucoma surgery, the patient developed erosion of the Ahmed glaucoma tube through conjunctiva and sclera graft. Sclera graft covering the tube melted, and 2 mm segment of tube was exposed (Fig. 1A). A revision surgery was planned.

### Surgical Technique

The epithelium around the erosion (6 mm in diameter) was removed by a blade (Fig. 1B). A 6 mm donor cornea patch graft was used to cover the tube. The patch graft was trimmed to create a thinned disk with beveled edges for a smoother graft-host interface; anticipating this would promote epithelialization. The patch graft was fixed in place by Tisseel tissue adhesive (Baxter AG, Vienna, Austria) (Figs 1C to E). Additionally, two 10-0 nylon sutures were placed to help keep the graft in place. The corneal graft was not covered by adjacent conjunctiva because it was deficient and dense scarring was present; conjunctiva could not be mobilized. The procedure was completed by performing nasal and temporal tarsorrhaphy. Postoperative management included a combination of antibiotic and steroid eyedrops.

## RESULTS

During 12 months after the surgery, the intraocular pressure (IOP) was maintained by latanoprost 0.005% eyedrops. Eye examination was performed frequently postoperatively, then every few months and included: IOP measurement (pneumotonometry), visual acuity measurement and slit-lamp examination. The IOP did not exceed 15 mm Hg. Visual acuity remained stable (20/200). No infections or other complications were recorded. Covering of the corneal patch graft by native conjunctival epithelium was noticed prior to the 1 month follow-up visit (Fig. 1F). At the last examination, the corneal graft remained securely in place and well epithelialized.

## DISCUSSION

For repair of an exposed tube, use of different patch graft materials have been described, such as autologous fascia lata, donor pericardium, sclera, cornea, acellular dermis graft, and bioengineered collagen implant.^8-13^ In our case, we used donor corneal graft for coverage of a revised glaucoma tube, as cornea may be the preferable material to provide stable tube coverage.^11^ Different methods to close conjunctival defects that may occur after glaucoma drainage device surgery have been reported. In certain situations, conjunctival tissue cannot be advanced to cover the graft. The use of amniotic membrane, free conjunctival graft or buccal tissue have been proposed to cover the conjunctival defect.^14-16^ Cornea used for transplant is known to be a reasonable substrate for re-epithelialization. Our case was remarkable for the large conjunctival deficit and fragile thin sclera, partially due to keratoglobus, as well as numerous previous surgeries. We wished to minimize suturing to avoid scleral injury. Under this circumstance, a minimally invasive technique was used to secure corneal patch, that eventually epithe-lialized. We observed that a corneal patch graft with a smooth transition zone to sclera could be secured with tissue adhesive and successfully re-epithelialized. The described technique may be particularly useful in eyes with scleromalacia and/or multiple conjunctival surgeries with scarring.

## CONCLUSION

Our case illustrates that primary conjunctival coverage of corneal patch graft, secured by tissue adhesive, is not mandatory. Conjunctival epithelium has the ability to cover the corneal patch during the first several weeks after surgery. This technique provides an additional surgical option for complex eyes with deficient sclera and conjunctiva.
